# Three Competitive ELISAs to Quantify the D-Antigen Content of Aluminum-Salt Adjuvanted Recombinant Polio VLPs (Types 1, 2, 3) to Enable Preformulation Characterization Studies

**DOI:** 10.3390/vaccines14060479

**Published:** 2026-05-28

**Authors:** Yanli Liu, John M. Hickey, Geetha Satya Sainaga Jyothi Vaskuri, Brandy Dotson, Sangeeta B. Joshi, David B. Volkin

**Affiliations:** Department of Pharmaceutical Chemistry, Vaccine Analytics and Formulation Center, University of Kansas, 2030 Becker Drive, Lawrence, KS 66047, USA

**Keywords:** vaccine, stability, formulation, adjuvant, preservatives, adju-phos, poliovirus, virus-like particles, ELISA, potency, D-antigen

## Abstract

Background/Objectives: Recombinant poliovirus (PV) virus-like particle (VLP) antigens mimic the conformation of the surface proteins in native PVs (i.e., serotype-specific D-antigen epitopes). Since they lack genomes and are non-infectious, PV-VLPs offer the promise of a safer, next-generation polio vaccine compared to traditional inactivated (IPV) or attenuated live (OPV) vaccines. Sandwich D-antigen ELISA formats are commonly used to measure the in vitro potency values (relative D-antigen content, DU/mL) of unadjuvanted trivalent IPV antigens. If IPV is formulated with aluminum-salt adjuvants, however, a pretreatment step (i.e., adjuvant dissolution or antigen desorption) is required, which may compromise antigen integrity during sample handling. Methods: This work describes the development of three competitive ELISAs to measure the relative D-antigen content of aluminum-salt adjuvanted PV-VLPs (Types 1, 2, 3) without the need for pretreatment. Results: First, key assay parameters were established, including specificity, accuracy, precision, linearity, limit of quantification, and stability-indication. Next, preformulation characterization studies were performed with these methods including (1) rank-ordering the inherent thermal stability profiles of the PV-VLPs (Types 1 > 3 > 2) in-solution and adsorbed to an aluminum phosphate adjuvant (AdjuPhos™, AP) and (2) determining the effect of formulation variables on the thermal stability profiles of AP-adsorbed PV-VLPs including antimicrobial preservatives (thimerosal, 2-PE) and five different antigens present in pediatric combination vaccines (D, T, wP, Hib, Hep B). Conclusions: The development and application of three competitive D-antigen ELISAs were demonstrated, and future use in formulation and storage stability studies with the AP-adjuvanted, trivalent PV-VLPs (Types 1, 2, 3) is discussed with the long-term goal to develop a stable, efficacious, multi-dose, hexavalent combination vaccine presentation.

## 1. Introduction

Poliovirus (PV), the causative agent of poliomyelitis (polio), belongs to the *Enterovirus coxsackiepol* species of the *Picornaviridae* family. PV is a highly infectious pathogen that primarily affects children under five years of age, leading to paralysis and, in the most severe cases, death [1,2]. For over six decades, two vaccines have been widely used to combat PV-associated morbidity and mortality: the attenuated oral PV vaccine (referred to as OPV or the Sabin PV vaccine) and the inactivated PV vaccine (referred to as IPV or the Salk PV vaccine). Since its launch in 1988, the Global Polio Eradication Initiative, coordinated by the World Health Organization (WHO), has reduced global polio cases by more than 99% through the global implementation of these safe and efficacious vaccines [3].

These two traditional PV vaccines have distinct advantages and limitations. OPV is a trivalent mixture of attenuated viruses (Types 1, 2, 3) and is inexpensive, easy to administer, and induces both humoral and intestinal mucosal immunity, making it highly effective in preventing viral transmission [4]. Due to intrinsic genetic instability, however, the attenuated PVs can lose attenuation and reacquire neurovirulence, leading to rare cases of circulating vaccine-derived polioviruses or vaccine-associated paralytic poliomyelitis. In contrast, IPV antigens cannot revert to an infectious form following formalin treatment; however, IPV manufacturing requires propagation of large quantities of virulent PVs prior to inactivation, presenting a potential biosafety risk and resulting in a more costly vaccine [5]. Trivalent IPV antigens (Types 1, 2, 3) are administered parenterally and formulated as either standalone or included in pediatric combination vaccines (for an in-depth review, see [6]). Most standalone IPV vaccines consist of a trivalent mixture of antigens in solution, while in combination pediatric vaccines, IPV is formulated in the presence of other antigens and aluminum-salt adjuvants. Although IPV induces strong systemic immune responses, mucosal immunity is more limited, making it less effective in blocking viral transmission [7].

Consequently, the development of safer, next-generation PV vaccine candidates is a priority in the post-eradication era, such as the recent development of poliovirus-like particles (PV-VLPs). In general, virus-like particles (VLPs) mimic the conformation of native pathogenic viruses, including antigens located on the viral capsid. Since they lack genomic and virulent components, they are non-infectious, enabling them to induce strong immunity while being safer to manufacture and administer compared to traditional viral vaccines (live or inactivated). The VLP-based vaccine platform has been utilized in many approved human vaccines, including those against human papillomavirus, hepatitis B/C/E viruses, coronavirus, chikungunya virus, and *Plasmodium* [8]. Since the VLP vaccine platform has demonstrated excellent efficiency and safety profiles, this is a compelling approach for a next-generation PV vaccine [9]. Currently, PV-VLP antigens of all three virulent serotypes (Types 1, 2, 3) have been successfully expressed and produced in common recombinant expression systems, including yeast, bacteria, plant, insect, and mammalian cells [10,11,12,13,14,15]. The effects of different expression systems (and the lack of formalin treatment) on the susceptibility of PV-VLPs to potency losses during manufacturing and storage remain to be determined, especially in comparison to IPV antigens. Both PV and PV-VLPs can undergo conformational changes that convert the native and protective immunity-inducing D-antigen epitopes into a nonprotective C-antigen epitope [16,17]. Since IPV vaccines are dosed based on the D-antigen content of each type, accurate analytical methodologies to quantify D-antigen content are required to ensure their efficacy and storage stability, and therefore, similar analytical procedures are required for PV-VLP-based vaccine candidates.

Both in vivo and in vitro analytical methods are available to measure the potency of IPV vaccines. For example, determining neutralizing antibody titers in rats is commonly employed as an in vivo potency method, while determining D-antigen content using D-antigen enzyme-linked immunosorbent assays (ELISA) is used as an in vitro potency assay. The two potency assay approaches have been shown to correlate (D-antigen levels with immunogenicity in animal models) and thus the D-antigen ELISA has been validated for use in vaccine process development and quality control [18,19,20,21]. The D-antigen ELISA assays utilize either monoclonal or polyclonal antibodies (Ab) to recognize one or more of the multiple D-antigen sites per PV protomer subunit in a standard sandwich format (i.e., one Ab to capture the PV antigen, while a second Ab is used for detection) [22]. One limitation of this D-antigen sandwich ELISA format is interference if the IPV antigens are adsorbed to aluminum-salt adjuvants, which are added in one standalone IPV vaccine and in multiple different IPV-containing pediatric combination vaccines. In these cases, dissolution of the aluminum-salt adjuvant or desorption of the antigen is typically required prior to analysis, and such a sample-handling step may compromise antigen integrity and assay accuracy [23,24,25]. The strength of interaction between antigens and aluminum-salt adjuvants can also strengthen/weaken during storage, making it more challenging to ensure consistent antigen recovery in the assay during stability studies [26].

To address these analytical limitations and to enable formulation development of PV-VLPs as a vaccine candidate, we developed three competitive ELISA methods capable of quantifying the D-antigen content of three PV-VLPs (Types 1,2,3) either as monovalent bulk samples (in solution) or as formulated drug product (adsorbed to aluminum phosphate adjuvant, Adju-Phos™ or AP), with the latter not requiring antigen desorption or adjuvant dissolution. Moreover, we utilized a single commercially monoclonal Ab for each PV type, making these three D-antigen competitive ELISAs more widely assessable for PV-VLP process and formulation development. The methods were evaluated for multiple analytical suitability parameters, including specificity, accuracy, precision, linearity, limit of quantification, and stability-indication. The applicability of the D-antigen competitive ELISAs was demonstrated by performing a series of preformulation characterization studies including examining the thermal stability profiles of each of the three PV-VLP antigens, both in monovalent and trivalent preparations, in the presence and absence of (1) aluminum-salt adjuvants, (2) two antimicrobial preservatives used in multi-dose vaccine formulations (Thimerosal (TH), 2-phenoxyethanol (2-PE)), and (3) five combination vaccine antigens used in pediatric combination vaccines administered in low- and middle-income countries (LMICs) (Diphtheria toxoid (D), Tetanus toxoid (T), Hepatitis B (Hep B), *Haemophilus influenza* type b (Hib), and whole-cell pertussis (wP)). In general, a hexavalent combination vaccine to protect against six diseases would be of interest globally. This particular work focused on the whole-cell pertussis antigen and on a multidose presentation used in LMICs with the goal of improving affordability, vaccine access, and patient compliance. The results demonstrate that the established D-antigen competitive ELISAs can be used to measure the relative in vitro potency levels of the three PV-VLPs under diverse formulation conditions, and these methods will facilitate the future formulation development of new PV-VLP vaccine candidates with the ultimate goal of developing a multidose, hexavalent pediatric combination vaccine targeted for use in LMICs.

## 2. Materials and Methods

### 2.1. Materials

Recombinant monovalent bulk PV-VLPs (Type 1, Type 2, and Type 3) were expressed in insect cells, purified, and provided as liquid bulks stored at 4 °C by CanSino Biologics (Tianjin, China) [27]. Both thimerosal-containing and thimerosal-free pediatric combination vaccine antigen bulks (D, T, Hep B, Hib, wP) were provided by Biofarma (Bandung, Indonesia) as liquid bulks stored at 4 °C. The preservatives TH and 2-PE were purchased from Sigma Aldrich (St. Louis, MO, USA). Adju-Phos^®^ adjuvant was purchased from InvivoGen (San Diego, CA, USA). Human monoclonal antibodies (mAb), 2D6 (20/250), 1B8 (20/252), and 6B5 (20/254), which are specific for a single D-antigen epitope on PV Type 1, Type 2, and Type 3, respectively, were purchased from the Medicines and Healthcare products Regulatory Authority (MHRA, Hertfordshire, UK). An HRP (Horseradish Peroxidase)-conjugated goat anti-human secondary antibody was purchased from Invitrogen (Carlsbad, CA, USA). All other reagents were purchased from commercial sources.

### 2.2. Methods

#### 2.2.1. Preparation of PV-VLPs Formulated with Aluminum Phosphate (AP) Adjuvant

The trivalent, AP-adjuvanted PV-VLP drug product (DP) formulations were prepared by mixing together the three PV-VLP bulks (Types 1, 2, 3) and then adsorbing them together to aluminum phosphate (AP, Adju-Phos™) adjuvant. Prior to adsorption, the pH of the bulk AP adjuvant in saline was adjusted to 6.0 using 1 N HCl. The three PV-VLPs (Type 1, 2, 3) bulks were diluted to 4× of their target concentrations (DU/mL), within an overall dilution range of 10–50-fold, with a buffer containing 10 mM Histidine, 150 mM NaCl, pH 5.0, to generate a trivalent bulk. The trivalent bulk was mixed thoroughly with 2.4 mg/mL AP (pH 6.0) by repeated pipetting and incubated at room temperature for 1 h without rotation to facilitate maximal adsorption. The 4× samples were diluted to the 1× target PV-VLP concentration for the DP with a final formulation buffer containing 20 mM Histidine, 150 mM NaCl, pH 6.0 such that the final target concentrations were 90 DU/mL for Type 1, 16 DU/mL for Type 2, and 50 DU/mL for Type 3 PV-VLPs, and 0.6 mg/mL AP in a final formulation buffer of 20 mM Histidine, 150 mM NaCl, pH 6.0. Non-adjuvanted trivalent PV-VLP formulations (in-solution) were prepared in parallel using the same procedure but without AP. All formulations were prepared and stored at room temperature in low-protein-binding tubes (Eppendorf, Hamburg, Germany).

For analysis in the D-antigen competitive ELISAs (see below), 0.5 mL of 1× DP sample stored in low-protein-binding tubes (Eppendorf) was centrifuged at 4000× *g* for 5 min, a 450 µL aliquot of supernatant was transferred to a new low-protein-binding tube, and the pellet was resuspended in 450 µL 20 mM Histidine, 150 mM NaCl, pH 6.0. The unfractionated (whole) sample, pellet, and supernatant were then analyzed using the corresponding PV type-specific D-antigen competitive ELISA described below.

#### 2.2.2. Thermal Stability Assessments of PV-VLPs Samples with Adjuvants, Antimicrobial Preservatives, and Five Combination Vaccine Antigens

The thermal stability of PV-VLP antigens, either in solution or adsorbed to AP adjuvant, was evaluated using PV type-specific competitive ELISAs. Trivalent PV-VLP antigens in-solution or AP-adsorbed were prepared in duplicate as described above, and the composition of the samples was 90 DU/mL (Type 1), 16 DU/mL (Type 2), and 50 DU/mL (Type 3) in 20 mM Histidine, 150 mM NaCl, pH 6.0 (in-solution) or adsorbed to 0.6 mg/mL AP. The samples were then subjected to heat treatment at 32, 35, 38, 41, 44, 47, 50, 53, 56, or 59 °C for 10 min. The relative in vitro potency of each heat-stressed sample was calculated as a percentage relative to the formulation-specific controls at 4 °C.

The thermal stability profiles of monovalent DP (Types 1, 2, 3 PV-VLPs adsorbed to AP) in the absence or presence of two preservatives, TH or 2-PE, were evaluated using the corresponding PV type-specific D-antigen competitive ELISA. Monovalent AP-adsorbed PV-VLP samples were prepared as described above with the inclusion of either TH (0.005% *w*/*v*) or 2-PE (0.5% or 1% *v*/*v*) during the 4× to 1× dilution step. Corresponding preservative-free monovalent PV-VLP samples served as controls. Individual samples in low-protein-binding tubes with a snap cap (Eppendorf) were subjected to heat stress for 10 min at 32, 35, 38, 41, 44, 47, 50, 53, 56, or 59 °C and then stored on ice. The relative in vitro potency values (DU/mL) of each heat-stressed sample and for their formulation-specific controls stored at 4 °C. Values of thermally stressed samples were then calculated as a percentage relative to the unstressed control by comparing the temperature at which the relative percent DU/mL values of each sample decreased by 50%.

The thermal stability profiles of trivalent DP (PV-VLPs adsorbed together to AP) were evaluated in the absence or presence of five combination vaccine antigens (D, T, Hib, Hep B, wP). A 4× trivalent PV-VLP DP was prepared as described above. A 2× combination vaccine antigen formulation was prepared by mixing D, T, Hib, Hep B, and wP antigens with AP adjuvant. Prior to adsorption, the pH of the bulk AP in saline was adjusted to 5.0 using 1 N HCl. The bulk combination antigens were diluted to 2× of their target concentrations (40 Lf/mL D, 40 Lf/mL T, 20 µg/mL Hib, 20 µg/mL Hep B, and 24 OU/mL wP), within an overall dilution range of 4-to-105-fold, with a buffer containing 10 mM Histidine, 150 mM NaCl, pH 5.0, and then thoroughly mixed with 1.32 mg/mL AP (pH 5.0). The 2× combination antigen formulations were stored at 4 °C overnight. As a control, a formulation was prepared with PV-VLPs using the same procedure, but without the five combination vaccine antigens. The following day, the pH of the 2× combination antigen formulation was adjusted to 6.0 with 50 mM Histidine, 150 mM NaCl, pH 6.4. The 4× trivalent PV-VLP DP was then mixed with either the 2× combination antigen formulation, or control formulation, to generate the 1× final formulations containing 90 DU/mL Type 1, 16 DU/mL Type 2, and 50 DU/mL Type 3 PV-VLPs, and 1.26 mg/mL AP in 20 mM Histidine, 150 mM NaCl, pH 6.0, in the presence or absence of 40 Lf/mL D, 40 Lf/mL T, 40 µg/mL Hib, 40 µg/mL Hep B, and 24 OU/mL wP. The samples prepared in low-protein-binding tubes (Eppendorf) were subjected to heat treatment for 10 min at 32, 35, 38, 41, 44, 47, 50, 53, 56, or 59 °C, and then stored on ice before testing. The relative in vitro potency values (DU/mL) for each heat-stressed sample were measured and then calculated as a percentage relative to those measured for the formulation-specific controls stored at 4 °C.

#### 2.2.3. Three Different PV-VLP Antigen (Types 1, 2, 3) Competitive ELISAs

Methodology—A D-antigen competitive ELISA was established for each PV-VLP (Type 1, Type 2, and Type 3) and then applied to quantify the D-antigen content of either monovalent bulks (in-solution) or trivalent drug products (adsorbed together to AP adjuvant). High-binding 96-well ELISA plates (Corning, Durham, NC, USA) were coated individually with 100 µL of one of the PV-VLPs (100 DU/mL Type 1, 6.25 DU/mL Type 2, or 50 DU/mL Type 3) diluted with DPBS (Cytiva, Wilmington, DE, USA) and then incubated overnight at 4 °C. On the same day, PV-VLP containing samples were prepared at 90 DU/mL (Type 1), 16 DU/mL (Type 2), and 50 DU/mL (Type 3), mixed with casein blocking buffer (casein containing 0.05% Tween-20, Thermo Fisher Scientific (Waltham, MA, USA)) at a 1:1 volumetric ratio, and incubated for 1 h at room temperature. The blocked samples were then transferred to the first row of 96-well PCR plates, and seven sequential serial dilutions were performed in casein blocking buffer. An equal volume of PV type-specific monoclonal Ab (purchased from MHRA; 20/250 for Type 1, 20/252 for Type 2, and 20/254 for Type 3) was added to the corresponding plates. PCR plates were sealed tightly with cap strips and rotated vertically overnight at 4 °C. The following day, ELISA plates were washed three times with 300 µL/well of PBST (PBS containing 0.05% Tween-20) and then blocked with 200 µL casein blocking buffer for 1 h at 25 °C. PCR plates were centrifuged at 1600× *g* for 3 min, and then 100 µL of supernatant was transferred to the corresponding ELISA plates (after washing with PBST to remove the casein blocking buffer) and incubated at 25 °C for 2 h (Type 1 and Type 2) or 18 h (Type 3). Next, the ELISA plates were washed with PBST, and 100 µL of goat anti-human secondary antibody (Invitrogen), diluted 1:5000 in casein blocking buffer, was added to each well and incubated at 25 °C for 1 h. The ELISA plates were washed with PBST, and then 100 µL of TMB substrate was added and incubated at room temperature in the dark for 10 min (Type 1 and Type 2) or 15 min (Type 3). The reaction was stopped with 50 µL of 1 N sulfuric acid, and the optical density at 450 nm (OD450nm) was measured within 5 min using a SpectraMax iD5 microplate reader and SoftMax Pro (v7.1) software (Molecular Devices, San Jose, CA, USA). Data were analyzed as described below.

Checkboard titration experiments—Checkerboard titrations were performed to determine the optimized levels of (1) each PV-VLP to coat the ELISA plates (i.e., coating concentration in DU/mL), and (2) dilutions of the corresponding serotype-specific D-antigen monoclonal antibody (i.e., mAb concentration in µg/mL). The titration procedures were similar for all three PV-VLPs and their corresponding serotype-specific D-antigen mAbs. For example, a Type 2 PV-VLP bulk sample was prepared at 100 DU/mL, serially diluted (100 to 0.78 DU/mL), and then added to the ELISA plates to coat them after overnight incubation. Plates were then washed and blocked, and serial dilutions of the Type 2 PV-specific D-antigen mAb were added (10 to 0.005 µg/mL). After incubation (2 h), detection was performed with an HRP-conjugated detection antibody and TMB reagent.

Data Analysis and Statistics—Data were analyzed in Origin (v2020) software (OriginLab Corporation, Northampton, MA, USA) using a four-parameter logistic (4-PL) curve fitting with no weighting to generate a standard curve of the PV-VLP reference DU/mL (freshly prepared monovalent or trivalent PV-VLP in-solution or adsorbed to AP for each experiment) vs. OD450nm values.

$$y=A2+\frac{A1-A2}{1+{\left(x/x0\right)}^{p}}$$

where *y* is the OD450nm value, *A*1 is the bottom plateau of the 4-PL curve, *A*2 is the top plateau of the 4-PL curve, *x*0 is the EC50 value, and *p* is the hill slope of the curve. *A*1, *A*2, and *p*-values from the reference were applied to fit the OD450 nm values of PV-VLPs in test samples using a 4-PL curve. The relative potency of each PV-VLP in a test sample relative to a reference was calculated as: Relative potency (%) = $\frac{x0\;of\;reference}{x0\;of\;test\;article}\times 100\%$. Data are reported as mean ± range (n = 2 replicates made independently and measured once) or as mean ± SD (n ≥ 3).

Accuracy and precision—Assay accuracy was evaluated by dilution recovery using three spiked levels (125%, 100%, and 75% of nominal concentration). For each level, three independent dilutions were prepared and tested in the same run. Accuracy was reported as recovery (%) and calculated as follows: Accuracy (%) = Observed relative potency/Expected value × 100. Inter-precision was assessed using 100% PV-VLP samples (Type 1: 90 DU/mL; Type 2: 16 DU/mL; Type 3: 50 DU/mL). Each sample was tested on different days by 2 or 3 analysts independently. Standard deviation (SD) and relative standard deviation (RSD) values were calculated. The acceptance criterion of inter-assay precision used in this work was an RSD of less than 20%, with no significant differences observed between repetitions.

Linearity and limit of quantification (LOQ)—These assay parameters were evaluated using both monovalent bulk PV-VLPs (in-solution) or trivalent drug products (adsorbed to AP). Samples were prepared by diluting each PV-VLP in a casein-blocking buffer to generate concentrations ranging from 125% to 5% of nominal values (90 DU/mL for Type 1, 16 DU/mL for Type 2, and 50 DU/mL for Type 3). D-antigen content of each dilution was measured using the corresponding PV type-specific D-antigen competitive ELISA. The criteria for acceptable linearity and LOQ were as follows: (1) coefficient of determination (R^2^) ≥ 0.95 for the 4-PL fit of the reference standard; (2) measured values within 80–120% of targeted concentrations; (3) RSD of ≤20%.

mAb Specificity and D-Antigen Stability Indication—To demonstrate the specificity of each commercial type-specific PV mAb, serotype-specific monovalent PV-VLP bulks, and bivalent antigen mixtures lacking the target PV-VLP, were prepared by diluting to 90 DU/mL (Type 1), 16 DU/mL (Type 2), and 50 DU/mL (Type 3) using their respective bulk formulation buffers (10 mM Histidine, 150 mM NaCl, 0.01% PS80, pH 6.7 for Types 1 and 2 PV-VLPs; 10 mM Sodium Phosphate, 150 mM NaCl, 0.01% PS80 pH 6.8 for Type 3). Each competitive PV-type-specific competitive ELISA was tested for cross-reactivity by comparing the measured D-antigen content of the bivalent mixtures against their corresponding monovalent PV-VLP controls. Stability-indication was established using monovalent PV-VLP bulks diluted to 90 DU/mL (Type 1), 16 DU/mL (Type 2), or 50 DU/mL (Type 3) in their respective bulk formulation buffers and aliquoted into 1.5 mL low-protein-binding tubes (Eppendorf) that were then incubated at 4 °C (unstressed control) or heat-stressed for 10 min at 38, 41, 44, 47, or 50 °C, and then analyzed using the corresponding PV type-specific D-antigen competitive ELISA.

## 3. Results and Discussion

### 3.1. Method Development of D-Antigen Competitive ELISAs

**Figure 1:**
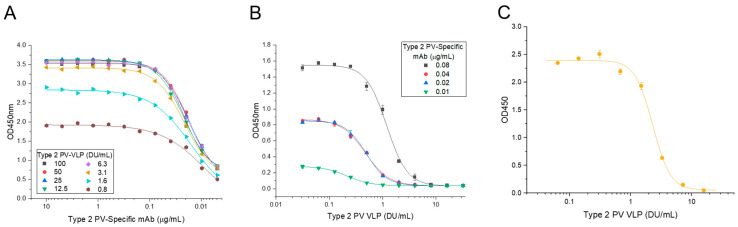
Method optimization of competitive ELISA conditions to determine relative D-antigen content of Type 2 PV-VLP bulk samples. (**A**) Representative checkerboard titration curves to optimize Type 2 PV-VLP coating concentration (100 to 0.78 DU/mL) and Type 2 specific D-antigen mAb dilutions (10 to 0.005 µg/mL). (**B**) Reactivity of Type 2 PV-VLP (32 to 0.03 DU/mL) in the presence of the Type 2 specific D-antigen mAb (0.08 to 0.01 µg/mL). (**C**) Representative 4-parameter logistic (4-PL) fit of antibody–antigen binding data for determining D-antigen content of a Type-2 PV-VLP control sample using the optimized experimental conditions for the competitive ELISA. Data denote the mean ± range from two independent replicates (two samples made independently and then each analyzed once).

Representative results are described below first for the monovalent bulk Type 2 PV-VLP sample. A plate coating concentration of 6.25 DU/mL of Type 2 PV-VLP was selected based on higher maximal OD values and minimal antigen usage (Figure 1A). The Type 2 PV-specific D-antigen mAb concentration was further optimized at different dilutions, and a 0.078 µg/mL concentration was selected due to the superior signal strength compared to the other concentrations while maintaining an archetypal sigmoidal curve (Figure 1B). Finally, the serial dilution factor for a Type 2 PV-VLP sample was adjusted to 2.2×, and the optimized D-antigen competitive ELISA was performed (i.e., with 6.25 DU/mL of Type 2 PV-VLPs to coat plates and 0.078 µg/mL of Type 2 PV-specific D-antigen mAb), resulting in a representative antigen–antibody binding curve as shown in Figure 1C.

Similar checkboard titration experiments were performed with the Type 1 and Type 3 PV-VLPs and their corresponding serotype-specific D-antigen mAbs. The optimized D-antigen competitive ELISA conditions were identified as follows (also see summary in Table 1): (i) for a Type 1 PV-VLP sample, serial dilutions were adjusted to 1.5×, with Type 1 PV-VLP plate coating at 100 DU/mL, and Type 1 PV specific D-antigen mAb concentration at 0.078 µg/mL, and (ii) for a Type 3 PV-VLP sample, serial dilutions were adjusted to 1.9×, with Type 3 PV-VLP plate coating at 25 DU/mL, and Type 3 PV specific D-antigen mAb concentration at 0.025 µg/mL.

### 3.2. Method Qualification for the Three D-Antigen Competitive ELISAs

#### 3.2.1. Specificity, Stability-Indication, Adjuvant Compatibility

The three D-antigen competitive ELISAs were designed to measure the relative in vitro potency (DU/mL value by comparison to a reference sample) of PV-VLPs (Types 1, 2, 3) either as monovalent bulks (in-solution) or trivalent drug product (DP) adsorbed to aluminum phosphate (AP, Adju-Phos™) adjuvant (see methods for DP composition and formulation process). For the DP samples, this competitive ELISA does not require desorption of the PV-VLPs or dissolution of the aluminum-salt adjuvant (see Introduction). To demonstrate that the three D-antigen competitive ELISAs were fit for their intended use (preformulation characterization and formulation development studies), we first established some key assay parameters, including specificity, stability-indication, and compatibility with AP adjuvant.

**Figure 2:**
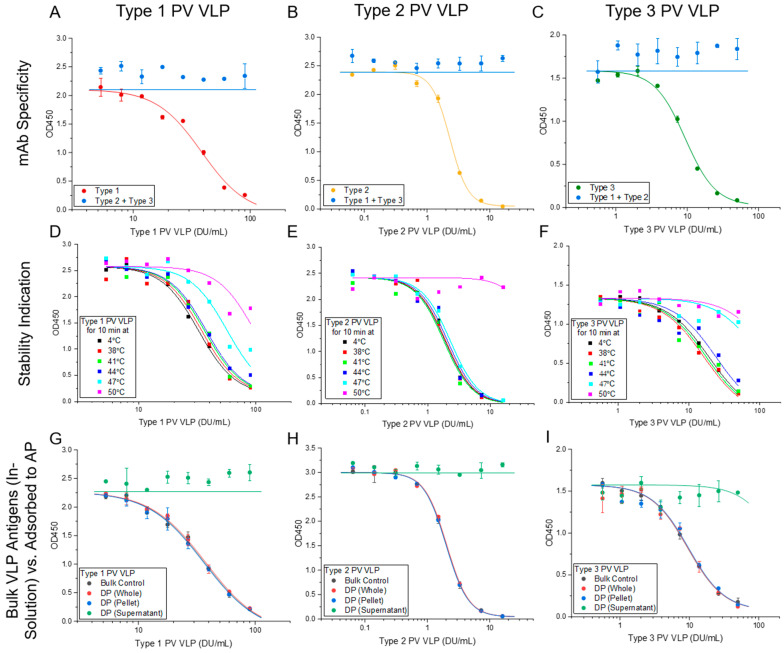
Specificity, stability-indication, and aluminum-salt adjuvant compatibility of the D-antigen competitive ELISAs for Types 1, 2, 3 PV-VLP samples. (**A**–**C**) Assay specificity for each PV-VLP bulk sample was demonstrated by comparing the binding curves of each serotype-specific PV mAb vs. serotype-specific PV-VLP vs. a mixture of the other two PV-VLPs. (**D**–**F**) Stability-indication (thermal stress) was demonstrated by incubating each monovalent bulk PV-VLP sample for either 10 min at 4 °C (unstressed control) or at the indicated elevated temperature values (range between 38 and 50 °C). Compositions of the monovalent bulk samples were as follows: Type 1 (90 DU/mL in 10 mM Histidine, 150 mM NaCl pH 6.7, 0.01% PS80), Type 2 (16 DU/mL in 10 mM Histidine, 150 mM NaCl pH 6.7, 0.01% PS80), and Type 3 (50 DU/mL in 10 mM Sodium Phosphate, 150 mM NaCl pH 6.8, 0.01% PS80) (**G**–**I**) Compatibility of the method with aluminum-salt adjuvants was evaluated by comparing antigen–antibody binding profiles of bulk trivalent PV-VLPs (in-solution) vs. formulated drug product (DP) PV-VLPs (mixed with colloidal suspension of aluminum phosphate adjuvant (Adju-phos™, AP) including unfractionated (whole) sample, and centrifuged sample (pellet and supernatant fractions)). Composition of the bulk (in-solution) sample was 90 DU/mL (Type 1), 16 DU/mL (Type 2), and 50 DU/mL (Type 3) in 20 mM Histidine, 150 mM NaCl pH 6.0 and composition of the formulated DP sample was 90 DU/mL (Type 1), 16 DU/mL (Type 2), and 50 DU/mL (Type 3) adsorbed together to 0.6 mg/mL AP in 20 mM Histidine, 150 mM NaCl pH 6.0. Data denote the mean ± range from two independent replicates.

First, the specificity of the serotype-specific PV D-antigen mAb was established by determining the ability to detect the corresponding serotype-specific PV-VLP in the presence of the other two PV-VLPs. As shown in Figure 2A–C, each serotype-specific D-antigen mAb selectively recognized its corresponding serotype-specific PV-VLP with no detectable cross-reactivity with the two other PV-VLPs. This result is consistent with the specificity properties previously reported for each of the three mAb reagents [28].

Next, stability-indication was evaluated for monovalent bulk PV-VLP samples at their target concentration (90, 16, and 50 DU/mL for Type 1, 2, and 3 PV-VLPs, respectively). Each PV-VLP sample was individually thermal-stressed (for 10 min at temperatures ranging from 38 to 50 °C), along with a control stored at 4 °C, and then assayed by the D-antigen competitive ELISAs (Figure 2D–F). The thermal-stressed PV-VLP samples exhibited notable decreases in mAb binding (i.e., increased mAb interaction with PV-VLPs coated on the ELISA plates) compared with unstressed control, particularly after incubation at ≥50 °C, as reflected by a progressive right-shift in the competitive ELISA curves as a function of increasing temperature. These data demonstrate the ability of each D-antigen competitive ELISA to detect degradation of each monovalent bulk PV-VLP sample.

Finally, to determine the compatibility with aluminum-salt adjuvants, the D-antigen competitive ELISA curves of each trivalent PV-VLP bulk (in-solution) were compared to trivalent PV-VLP drug product (DP) samples (adsorbed to the aluminum phosphate adjuvant Adju-Phos™, AP). The DP samples were tested either unfractionated (whole) or after centrifugation (pellet and supernatant fractions due to the colloidal nature of the AP adjuvant). As shown in Figure 2G–I, the D-antigen competitive ELISA curves of each monovalent bulk PV-VLP sample were comparable to both the whole DP sample and the pellet fraction, while no-to-little mAb binding in the supernatant fraction. These results indicate (1) each PV-VLP was ~100% adsorbed to the AP adjuvant, and (2) D-antigenicity values were similar between bulk (in-solution) and DP (adsorbed to AP adjuvant) PV-VLP samples.

#### 3.2.2. Accuracy, Precision, Linearity, LOQ

As the next step to demonstrate that the three D-antigen competitive ELISAs were fit for their intended use (preformulation characterization and formulation development studies), we evaluated key assay parameters for method qualification, including accuracy, precision, linearity, and limit of quantification (LOQ). These values were established for both the monovalent bulks and the AP-adjuvanted trivalent DP for the Types 1, 2, 3 PV-VLPs, with a summary of results provided in Table 1 and described below.

The accuracy and precision values of the three D-antigen competitive ELISAs were evaluated at the target PV-VLP concentrations (90 DU/mL for Type 1, 16 DU/mL for Type 2, and 50 DU/mL for Type 3) for both monovalent bulk (in-solution) and trivalent DP (AP-adsorbed) samples. Two or three analysts independently performed the competitive ELISAs. As shown in Table 1, the measured values were within 10% of their expected target values (accuracy). Across the different PV-VLPs and sample types, relative standard deviation values (RSD) were below 15%, with an overall average precision of 6% with the monovalent bulk and 7–11% in the trivalent DP samples (precision). The difference in replicate numbers in Table 1 reflects the availability of each bulk material during method development and preformulation characterization studies. Monovalent bulk samples were evaluated in earlier stages of assay development and thus with fewer replicates, while AP-adsorbed samples were tested during later stages of assay qualification and during preformulation characterization studies and thus with higher replicate numbers.

For linear range and LOQ, both monovalent bulk PV-VLP (in-solution) and trivalent PV-VLP DP (adsorbed to AP) samples were tested across 125–5% of their target concentrations. For Type 1 PV-VLP, linear responses were measured for the monovalent bulk and trivalent DP between 18 and 113 DU/mL and 22.5–113 DU/mL, respectively. For Type 2 PV-VLP, a linear response range of 2.4–20 DU/mL was observed for both bulk and DP samples. Finally, for the Type 3 PV-VLPs, linear ranges were established as 12.5–62.5 DU/mL and 15–62.5 DU/mL for the monovalent bulk and trivalent DP samples, respectively. The LOQ was defined as the lowest concentration within each D-antigen competitive ELISA linear range with a measured accuracy of 80–120% and precision of ≤20% (see Supplementary Tables S1–S6). The LOQ values of the trivalent DP samples trended higher compared to the monovalent bulk samples for two of three PV-VLP samples (18 vs. 22.5 DU/mL for Type 1 and 12.5 vs. 15 DU/mL for Type 3), while values for Type 2 were similar (2.4 DU/mL for both samples).

In summary, method qualification results demonstrate that the three D-antigen competitive ELISAs exhibited suitable specificity, accuracy, precision, linearity, LOQ, and stability indication for use as a relative in vitro potency of PV-VLP (Types 1, 2, and 3) to facilitate initial formulation development work (i.e., preformulation characterization studies) as described in the next section.

### 3.3. Preformulation Characterization of PV-VLPs (Types 1, 2, 3)

#### 3.3.1. Inherent Thermal Stability Profiles with and Without Aluminum-Salt Adjuvant

As a first application of the D-antigen competitive ELISAs, we evaluated and compared the inherent thermal stability of the three PV-VLPs (Types 1, 2, 3) both in solution and after adsorption to AP-adjuvant. The goals of these preformulation characterization experiments were to (1) rank-order their stability profiles (loss of D-antigen activity vs. temperature) and (2) evaluate the effect of antigen adsorption to aluminum-salt adjuvants, since it has been previously reported that structural alterations of antigens can occur, leading to more or less epitope exposure, reduced [29] or enhanced stability [30] (e.g., vs. temperature or proteolytic degradation) and enhanced [31] or unaffected [32] in vivo animal immunogenicity [26,33].

**Figure 3:**
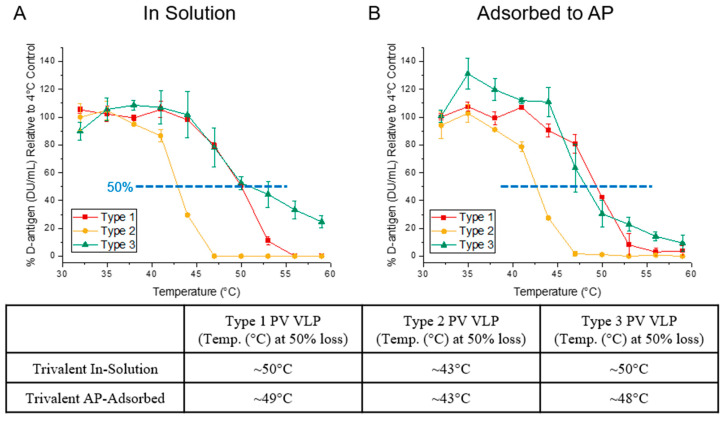
Rank-ordering the inherent thermal stability profiles of the Types 1, 2, 3 PV-VLPs (in-solution and bound to AP-adjuvant) using the D-antigen competitive ELISA assays. Thermal stability profiles of trivalent PV-VLPs (**A**) in-solution or (**B**) adsorbed together to AP adjuvant following 10 min incubation at the indicated temperature (range between 32 and 59 °C) and plotted as relative D-antigen content (i.e., DU/mL value at each temperature normalized to DU/mL value for each formulation-specific control at 4 °C). The approximate temperature values for each sample at which the relative DU/mL decreased by 50% (blue dashed line) are summarized in the accompanying table. Formulation composition was 90 DU/mL (Type 1), 16 DU/mL (Type 2), and 50 DU/mL (Type 3) in 20 mM Histidine, 150 mM NaCl, pH 6.0 for the monovalent bulk (in-solution) and trivalent drug product (adsorbed together to 0.6 mg/mL AP). Data denote the mean ± range from two independent replicates.

Thermal stability profiles of trivalent PV-VLPs either in solution or adsorbed to AP were determined at their target dose concentrations (90, 16, and 50 DU/mL for Type 1, 2, and 3 PV-VLPs, respectively). Each PV-VLP sample was thermal-stressed for 10 min at temperatures ranging from 32 to 59 °C, with a non-stressed control stored at 4 °C, followed by analysis using D-antigen competitive ELISAs (Figure 3A,B). Across all three serotypes, comparable thermal stability profiles were observed between each PV-VLP in-solution and adsorbed to AP (≤2 °C difference), e.g., a 50% loss of DU/mL occurred in Type 1, 2, or 3 at 49–50 °C, ~43 °C, or 48–50 °C, respectively. For both PV and PV-VLPs, it has been reported that a conversion from D-antigen (protective immunity-inducing epitopes) to C-antigen (non-protective epitopes) occurs between 33 and 62 °C, which is consistent with the PV-VLPs in this study undergoing a similar conformational change at these temperatures [11,12,14,34].

Rank-ordering of the inherent thermal stability profiles of these three PV-VLPs indicated that Type 1 was the most stable, followed closely by Type 3, and Type 2 was the least stable. Different thermal stability rank-order profiles have been reported between recombinant PV-VLP serotypes produced from insect cells in the literature, and such variations are not unexpected due to numerous experimental factors different between the studies, which include amino acid composition of the PV proteins, formulation conditions, and thermal-stress parameters. For example, a recent study by Sherry et al. (2025) demonstrated that insect cell-expressed Type 3 VLPs exhibited the greatest thermal stability, followed by Type 2, and Type 1 was the least stable [14]. By contrast, another study by Xu et al. (2019) showed that non-adjuvanted PV-VLP Type 1 was the most stable during a 24 d incubation at 37 °C, followed by Type 2 and then Type 3 [13].

In terms of future preformulation characterization studies with these three PV-VLPs (Types 1, 2, 3) using the three D-antigen competitive ELISAs, parallel stress stability experiments with physicochemical measurements (e.g., purity by SDS-PAGE and LC-MS and particle size by DLS and TEM) and/or with immunological readouts (e.g., animal immunogenicity) are suggested. For the former, correlations between physicochemical changes and the loss of D-antigenicity during stress would provide insights into the molecular mechanisms of PV-VLP degradation pathways, which could guide the development of stabilized formulations. For the latter, correlations between in vivo immunogenicity and loss of D-antigenicity would assess the sensitivity of in vivo vs. in vitro potency assay to structural changes within the PV-VLPs. As an example, it has been reported that the D-antigen assay is more sensitive than the rat potency assay to detect degradation of inactivated polio antigens [35]. In addition, such forced degradation studies would help to identify the critical quality attributes of the recombinant PV-VLP antigens to support future process and product development, as well as comparability studies [36,37].

#### 3.3.2. Effect of Antimicrobial Preservatives

Next, we evaluated the utility of the three D-antigen competitive ELISAs to determine the effect of antimicrobial preservatives on the thermal stability of AP-adsorbed trivalent PV-VLPs (Types 1, 2, 3). The goal was to assess the feasibility of developing a lower-cost, multidose vaccine formulation targeted for use in LMICs by providing multiple doses in a single vial, which lowers overall cost by reducing costs associated with packaging, labeling, medical waste generation, cold-chain storage space requirements, and transportation [38]. Two preservatives were examined in this work: (1) thimerosal (TH), which has historically been used in combination vaccines containing whole-cell pertussis antigen, and (2) 2-phenoxyethanol (2-PE), which is used in licensed multidose IPV containing vaccines [6,39]. While TH is known to be incompatible with IPV [40], TH compatibility with recombinant PV-VLPs has not been reported to the best of our knowledge. Commonly used concentrations of TH (0.005% *w*/*v*) and 2-PE (0.5% and 1% *v*/*v*) were evaluated to mimic levels reported in some marketed vaccines [41].

**Figure 4:**
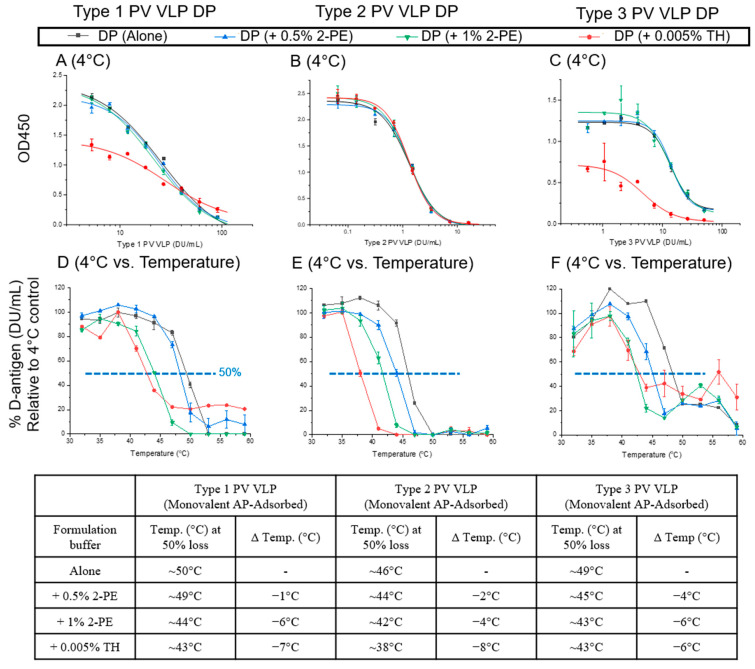
Effect of antimicrobial preservatives on thermal stability profiles of monovalent AP-adjuvanted PV-VLPs (Types 1, 2, 3) as measured by D-antigen competitive ELISAs. Representative competitive ELISA curves of unstressed (4 °C) monovalent Type 1 (**A**), Type 2 (**B**), or Type 3 (**C**) PV-VLPs adsorbed individually to AP adjuvant in formulation buffer alone (no preservative, black line), +0.5% 2-PE (blue line), +1% 2-PE (green line), or +0.005% TH (red line). Thermal stability profiles of AP-adsorbed monovalent PV-VLPs Type 1 (**D**), Type 2 (**E**), or Type 3 (**F**) following 10 min incubation at the indicated temperature (range between 32 and −59 °C) and plotted as relative D-antigen content (DU/mL value at each temperature normalized to DU/mL value for each formulation-specific control at 4 °C). The approximate temperature values for each sample at which the relative DU/mL decreased by 50% (blue dashed line) are summarized in the accompanying table. Composition of the formulated monovalent drug product (DP) sample was 90 DU/mL (Type 1), 16 DU/mL (Type 2), or 50 DU/mL (Type 3) adsorbed together to 0.6 mg/mL AP in 10 mM Histidine, 150 mM NaCl pH 6.0 in the absence or presence 0.5% 2-PE, 1% 2-PE, or 0.005% TH. AP, aluminum phosphate (Adju-phos™). Data denote the mean ± range from two independent replicates.

At 4 °C, the D-antigen competitive ELISA curves of the TH-containing AP-adsorbed trivalent PV-VLP samples were notably distinct compared to each of the no preservative control and the two 2-PE formulations (Figure 4A–C). For example, the maximal OD450 values were lower, and the slopes of the 4-PL fits were different in Type 1 and Type 3 TH-containing samples. These observations indicate that the TH rapidly impacted the Type 1 and Type 3 PV-VLPs used to coat the ELISA plates (and/or their related mAbs). Given this instability, a formulation-specific control held at 4 °C was used to calculate the relative in vitro potency values of each formulation as a function of temperature.

As shown in Figure 4D–F, for each AP-adsorbed trivalent PV-VLP sample, decreasing DU/mL values were observed with increasing temperature. Using the temperature value associated with a 50% decrease in DU/mL values (vs. the 4 °C control), the thermal stability of each AP-adsorbed trivalent PV-VLP sample decreased similarity across the three PV-VLPs in the presence of either antimicrobial preservative with 0.005% TH exerting the largest destabilizing effect (6–8 °C decrease), followed by 1% 2-PE (4–6 °C), then 0.5% 2-PE (1–4 °C).

These results demonstrate (1) the addition of preservatives, particularly 0.005% TH or 1% 2-PE, compromised the thermal stability of all three AP-adsorbed PV-VLPs, (2) the 2-PE-induced instability was concentration-dependent, (3) PV-VLPs display similar instability to TH as has been previously reported for IPV, while the relative sensitivity of PV-VLP vs. IPV to 2-PE remains to be determined. While these observations complicate the development of a multi-dose vaccine formulation containing AP-adsorbed trivalent PV-VLPs, multi-dose formulation development studies are ongoing to identify stabilizing excipients and optimized solution conditions to mitigate preservative-induced instability. These studies use the D-antigen competitive ELISAs to monitor the storage stability of AP-adsorbed trivalent PV-VLPs at different temperatures and under different formulation conditions, in the presence of various concentrations and types of preservatives.

#### 3.3.3. Effect of Combination Vaccine Antigens

Trivalent IPV vaccine formulations have diversified from standalone presentations to lower-cost pediatric combination vaccines that protect against multiple diseases while reducing the number of immunizations required for LMICs [42]. IPV-containing combination vaccines reduce overall cost by simplifying immunization schedules and lowering programmatic expenses, including the use of syringes, reduced cold-chain storage, and transportation requirements [43]. To investigate if the D-antigen competitive ELISAs could be used to develop pediatric combination vaccine formulations containing these PV-VLPs, five combination vaccine antigens (Diphtheria toxoid (D), Tetanus toxoid (T), Hepatitis B (Hep B), *Haemophilus influenza* type b (Hib), and whole-cell pertussis (wP)) were mixed with AP-adsorbed trivalent PV-VLPs.

**Figure 5:**
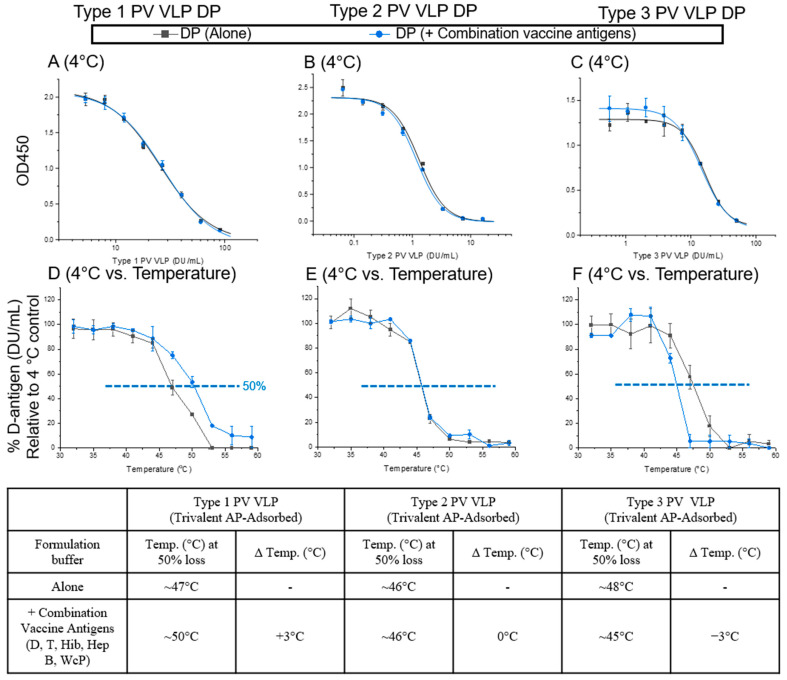
Effect of pentavalent mixture of pediatric combination vaccine antigens (D, T, Hib, Hep B, wP) on thermal stability profiles of trivalent AP-adjuvanted PV-VLPs (Types 1, 2, 3) as measured by D-antigen competitive ELISAs. Representative competitive ELISA curves of unstressed (4 °C) trivalent Type 1 (**A**), Type 2 (**B**), or Type 3 (**C**) PV-VLPs adsorbed together to AP adjuvant in formulation buffer alone (black line) and in the presence of five pediatric combination vaccine antigens (D, T, Hib, Hep B, wP) (blue line). Thermal stability profiles of AP-adsorbed trivalent PV-VLPs Type 1 (**D**), Type 2 (**E**), or Type 3 (**F**) following 10 min incubation at the indicated temperature and plotted as relative D-antigen content (i.e., DU/mL value at each temperature normalized to DU/mL value for each formulation-specific control at 4 °C). The approximate temperature values for each sample at which the relative DU/mL decreased by 50% (blue dashed line) are summarized in the accompanying table. Composition of the formulated trivalent drug product (DP) was 90 DU/mL (Type 1), 16 DU/mL (Type 2), and 50 DU/mL (Type 3) adsorbed together to 0.6 mg/mL AP in 20 mM Histidine, 150 mM NaCl pH 6.0 in the presence of 0.66 mg/mL AP alone (black traces) or premixed with 40 Lf/mL D, 40 Lf/mL T, 20 µg/mL Hib, 20 µg/mL Hep B, and 24 OU/mL wP (blue traces). AP, aluminum phosphate (Adju-phos™). Data denote the mean ± range from two independent replicates.

As shown in Figure 5A–C, no differences were noted in the D-antigen competitive ELISA curves of each PV-VLP (Types 1, 2, 3) at 4 °C in the presence or absence of the pentavalent mixture of combination vaccine antigens, indicating no impact of the five antigens on either (1) the D-antigen competitive ELISA itself, and (2) the short-term compatibility of the AP-adsorbed PV-VLPs with the pentavalent antigens.

The AP-adsorbed trivalent PV-VLPs, in the presence or absence of the pentavalent mixture combination antigens, were incubated for 10 min at temperatures ranging from 32 to 59 °C, and the relative in vitro potency values of each sample were compared to the formulation-specific controls held at 4 °C (Figure 4D–F). First, for thermal stability profiles of the three AP-adsorbed PV-VLPs alone (no combination antigens added), although results were overall similar to the control samples shown in Figure 4 (i.e., no preservatives added), some day-to-day variability was noted. For example, for the Type 1 PV-VLP, the 50% decrease temperature value was 47 °C vs. 50 °C, a result highlighting the importance of including a PV-VLP-alone control in every separate experiment. In the presence of the five combination vaccine antigens, the PV-VLPs exhibited distinct type-specific thermal stability profiles for the AP-adsorbed trivalent sample. For example, while the thermal stability profile of the Type 2 PV-VLP component was unaffected by the presence of the five combination vaccine antigens, the Type 1 PV-VLP displayed improved thermal stability, while the Type 3 PV-VLP showed decreased thermal stability (by ~3 °C).

The underlying mechanism(s) imparting the observed PV-VLP stability/instability profiles in the presence of the five combination vaccine antigens are unknown and are a focus of ongoing studies. Although these observations complicate the development of a formulation containing AP-adsorbed trivalent PV-VLPs and the five different combination vaccine antigens, ongoing combination vaccine formulation development studies are aimed at mitigating destabilizing effects by identifying stabilizing excipients and optimized solution conditions. The D-antigen competitive ELISAs are the key assays used to monitor the storage stability of AP-adsorbed trivalent PV-VLPs at different temperatures in different formulations in the presence of the combination vaccine antigens.

## 4. Conclusions

In this work, we developed and qualified three D-antigen competitive ELISAs to measure the relative in vitro potency values of recombinant PV-VLPs (Type 1, Type 2, Type 3) either as monovalent bulks (in solution) or trivalent drug products (adsorbed to aluminum phosphate adjuvant). The three D-antigen competitive ELISAs were shown to be specific for each PV-VLP serotype, stability-indicating, and compatible with aluminum-salt adjuvants. Method qualification results established accuracy, precision, linearity, and LOQ values and demonstrated that the methods are fit-for-use for preformulation characterization and formulation development studies. To this end, the thermal stability profiles for each PV-VLP serotype (i.e., relative in vitro potency values vs. increasing temperature exposure compared to the unstressed control) were successfully evaluated in a series of preformulation characterization studies. We rank-ordered the inherent thermal stability of each PV-VLP type (in-solution and adsorbed to AP-adjuvant) and elucidated the effect of antimicrobial preservatives and five combination vaccine antigens (D, T, Hib, Hep B, wP) on the thermal stability profiles of trivalent, AP-adsorbed PV-VLPs.

These studies not only demonstrated the analytical capabilities of the three D-antigen competitive ELISAs, but also established them as key methods for ongoing formulation development studies including (1) screening excipients to identify more stable formulations to better mitigate observed instability of the AP-adsorbed PV-VLPs, and (2) assessing the extent of correlations (if any) between the experimental stressed thermal studies reported in this work vs. storage stability studies in pharmaceutical containers (e.g., incubation at 2–8 °C and 25 °C over months and years), especially in the context of the determining the effects of preservatives (i.e., multidose formulations) and addition of other antigens (i.e., combination vaccines) on the stability profiles of AP-adsorbed PV-VLPs.

## Figures and Tables

**Table 1:** Summary of D-antigen competitive ELISA method qualification parameters for PV-VLP Types 1, 2, 3, as determined for monovalent bulks (in-solution) and trivalent drug product (adsorbed together to AP adjuvant). Formulation compositions of the monovalent bulks were Type 1 (90 DU/mL), Type 2 (16 DU/mL and Type 3 (50 DU/mL) in 10 mM buffer (sodium phosphate or histidine), 150 mM NaCl, 0.01% PS80 at pH 6.8. The trivalent drug product was similar but included 0.6 mg/mL AP (Adju-phos™) in a 20 mM histidine and 150 mM NaCl at pH 6.0. buffer. All values are presented as mean ± SD (n ≥ 3 with n values shown in Table).

	Type 1 PV-VLP		Type 2 PV-VLP		Type 3 PV-VLP	
Composition	Monovalent Bulk (In-Solution)	Trivalent Drug Product (AP-Adsorbed)	Monovalent Bulk (In-Solution)	Trivalent Drug Product (AP-Adsorbed)	Monovalent Bulk (In-Solution)	Trivalent Drug Product (AP-Adsorbed)
Target D-antigen concentration	90 DU/mL		16 DU/mL		50 DU/mL	
Serial Dilution Factor	1.5×		2.2×		1.9×	
Accuracy ± Precision (% Target D-antigen concentration)	95 ± 6 (n = 9)	99 ± 7 (n = 18)	96 ± 6 (n = 6)	106 ± 10 (n = 18)	109 ± 6 (n = 3)	104 ± 11 (n = 33)
Linear Range	18–113 DU/mL	22.5–113 DU/mL	2.4–20 DU/mL	2.4–20 DU/mL	12.5–62.5 DU/mL	15–62.5 DU/mL
Linear Range Slope (measured vs. expected D-antigen concentration %) and (r^2^ of Linear Regression Fit)	0.97 (≥0.99)	1.03 (≥0.99)	0.97 (≥0.99)	0.99 (≥0.99)	1.11 (≥0.99)	1.05 (≥0.99)
Estimated Limit of Quantification	18 DU/mL	22.5 DU/mL	2.4 DU/mL	2.4 DU/mL	12.5 DU/mL	15 DU/mL

## Data Availability

The datasets generated and/or analyzed in this study and Supplemental Information are available online in the KU ScholarWorks repository, https://doi.org/10.17161/1808.36357.

## Supplementary Materials

The following supporting information can be downloaded at: https://www.mdpi.com/article/10.3390/vaccines14060479/s1, Table S1. Linearity and limit of quantification results for monovalent bulk Type 1 PV-VLP. Table S2. Linearity and limit of quantification results for monovalent bulk Type 2 PV-VLP. Table S3. Linearity and limit of quantification results for monovalent bulk Type 3 PV-VLP. Table S4. Linearity and limit of quantification results for AP-adjuvanted trivalent PV-VLPs (Types 1, 2, and 3 adsorbed together to AP) using the Type 1 Competitive ELISA. Table S5. Linearity and limit of quantification results for AP-adjuvanted trivalent PV-VLPs (Types 1, 2, and 3 adsorbed together to AP) using the Type 2 Competitive ELISA. Table S6. Linearity and limit of quantification results for AP-adjuvanted trivalent PV-VLPs (Types 1, 2, and 3 adsorbed together to AP) using the Type 3 Competitive ELISA.

## Abbreviations

2-PE	2-Phenoxyethanol
4PL	Four-Parameter Logistic
Ab	Antibody
AP	Adju-Phos™
BCA	Bicinchoninic Acid
D	Diphtheria Toxoid
DLS	Dynamic Light Scattering
DP	Drug Product
ELISA	Enzyme-Linked Immunosorbent Assay
Hep B	Hepatitis B
Hib	*Haemophilus influenza* type b
HRP	Horseradish Peroxidase
IPV	Inactivated Polio Vaccine
LOQ	Limit of Quantification
mAb	Monoclonal Antibody
MHRA	Medicines and Healthcare Products Regulatory Authority
OD450nm	Optical Density at 450 nm
OPV	Oral Polio Vaccine
PV	Poliovirus
RSD	Relative Standard Deviation
SD	Standard Deviation
SDSPAGE	Sodium Dodecyl Polyacrylamide Gel Electrophoresis
T	Tetanus Toxoid
TEM	Transmission Electron Microscopy
TH	Thimerosal
VLP	Virus-like particle
wP	Whole-Cell Pertussis

## References

[1] Minor P. (2014). The polio endgame. Hum. Vaccines Immunother..

[2] Racaniello V.R. (2006). One hundred years of poliovirus pathogenesis. Virology.

[3] Global Polio Eradication Initiative Update from the Global Polio Eradication Initiative on Programmatic Challenges and Accountability. https://polioeradication.org/.

[4] Bandyopadhyay A.S., Burke R.M., Hawes K.M. (2024). Polio Eradication: Status, Struggles and Strategies. Pediatr. Infect. Dis. J..

[5] Liang J., Zhang Q., Li Y., Wang L. (2025). Advances and challenges in poliomyelitis vaccines: A comprehensive review of development, production, and global deployment. Front. Public Health.

[6] Kumar P., Bird C., Holland D., Joshi S.B., Volkin D.B. (2022). Current and next-generation formulation strategies for inactivated polio vaccines to lower costs, increase coverage, and facilitate polio eradication. Hum. Vaccines Immunother..

[7] Hird T.R., Grassly N.C. (2012). Systematic review of mucosal immunity induced by oral and inactivated poliovirus vaccines against virus shedding following oral poliovirus challenge. PLoS Pathog..

[8] Bachmann M.F., van Damme P., Lienert F., Schwarz T.F. (2025). Virus-like particles: A versatile and effective vaccine platform. Expert Rev. Vaccines.

[9] Mohsen M.O., Bachmann M.F. (2022). Virus-like particle vaccinology, from bench to bedside. Cell. Mol. Immunol..

[10] Bahar M.W., Porta C., Fox H., Macadam A.J., Fry E.E., Stuart D.I. (2021). Mammalian expression of virus-like particles as a proof of principle for next generation polio vaccines. npj Vaccines.

[11] Hong Q., Wang S., Wang X., Han W., Chen T., Liu Y., Cheng F., Qin S., Zhao S., Liu Q. (2024). Vaccine Potency and Structure of Yeast-Produced Polio Type 2 Stabilized Virus-like Particles. Vaccines.

[12] Marsian J., Fox H., Bahar M.W., Kotecha A., Fry E.E., Stuart D.I., Macadam A.J., Rowlands D.J., Lomonossoff G.P. (2017). Plant-made polio type 3 stabilized VLPs-a candidate synthetic polio vaccine. Nat. Commun..

[13] Xu Y., Ma S., Huang Y., Chen F., Chen L., Ding D., Zheng Y., Li H., Xiao J., Feng J. (2019). Virus-like particle vaccines for poliovirus types 1, 2, and 3 with enhanced thermostability expressed in insect cells. Vaccine.

[14] Sherry L., Bahar M.W., Porta C., Fox H., Grehan K., Nasta V., Duyvesteyn H.M.E., De Colibus L., Marsian J., Murdoch I. (2025). Recombinant expression systems for production of stabilised virus-like particles as next-generation polio vaccines. Nat. Commun..

[15] Han T., Xiao J., Zhang S., Su T., Liu Y., Zhang Y. (2026). Research Progress Towards Poliovirus Virus-like Particle Vaccines: A Review. Vaccines.

[16] Le Bouvier G.L. (1955). The modification of poliovirus antigens by heat and ultraviolet light. Lancet.

[17] Sherry L., Grehan K., Swanson J.J., Bahar M.W., Porta C., Fry E.E., Stuart D.I., Rowlands D.J., Stonehouse N.J. (2022). Production and Characterisation of Stabilised PV-3 Virus-like Particles Using Pichia pastoris. Viruses.

[18] Wilton T., Martín J. (2016). Methods for the Quality Control of Inactivated Poliovirus Vaccines. Poliovirus. Methods in Molecular Biology.

[19] Wood D.J., Heath A.B., Sawyer L.A. (1995). A WHO Collaborative study on assays of the antigenic content of inactivated poliovirus vaccines. Biologicals.

[20] Wood D.J., Heath A.B. (1995). A WHO collaborative study of immunogenicity assays of inactivated poliovirus vaccines. Biologicals.

[21] (2015). Recommendations to assure the quality, safety and efficacy of poliomyelitis vaccines (inactivated) Replacement of Annex 2 of WHO Technical Report Series, No. 910. WHO Tech. Rep. Ser..

[22] Kouiavskaia D., Puligedda R.D., Dessain S.K., Chumakov K. (2020). Universal ELISA for quantification of D-antigen in inactivated poliovirus vaccines. J. Virol. Methods.

[23] Rinella J.V., White J.L., Hem S.L. (1998). Effect of pH on the Elution of Model Antigens from Aluminum-Containing Adjuvants. J. Colloid Interface Sci..

[24] Seeber S.J., White J.L., Hem S.L. (1991). Solubilization of aluminum-containing adjuvants by constituents of interstitial fluid. PDA J. Pharm. Sci. Technol..

[25] Zhou X., Hu P., Lv H., Zhao L., Li J., Wang Y., Xie L. (2024). Desorption Kinetics Characterization of Virus-Like Particles in Polyvalent HPV Vaccine Containing Aluminum Phosphate Adjuvant. Am. J. Biomed. Sci. Res..

[26] HogenEsch H., O’Hagan D.T., Fox C.B. (2018). Optimizing the utilization of aluminum adjuvants in vaccines: You might just get what you want. npj Vaccines.

[27] Huang N., Wei Y., Li J. (2025). Insect cell expression system: Advances in applications, engineering strategies, and bioprocess development. J. Biol. Eng..

[28] (2022). Report on the WHO Collaborative Study to Establish Universal Reagents for the D-Antigen Potency Testing of Inactivated Polio Vaccines.

[29] Iyer V., Hu L., Liyanage M.R., Esfandiary R., Reinisch C., Meinke A., Maisonneuve J., Volkin D.B., Joshi S.B., Middaugh C.R. (2012). Preformulation characterization of an aluminum salt-adjuvanted trivalent recombinant protein-based vaccine candidate against Streptococcus pneumoniae. J. Pharm. Sci..

[30] Colaprico A., Senesi S., Ferlicca F., Brunelli B., Ugozzoli M., Pallaoro M., O’Hagan D.T. (2020). Adsorption onto aluminum hydroxide adjuvant protects antigens from degradation. Vaccine.

[31] Wang Z.B., Li S.X., Shan P., Wei D.Q., Hao S.J., Zhang Z., Xu J. (2022). Improved Aluminum Adjuvants Eliciting Stronger Immune Response When Mixed with Hepatitis B Virus Surface Antigens. Acs Omega.

[32] Bajoria S., Kaur K., Kumru O.S., Van Slyke G., Doering J., Novak H., Rodriguez Aponte S.A., Dalvie N.C., Naranjo C.A., Johnston R.S. (2022). Antigen-adjuvant interactions, stability, and immunogenicity profiles of a SARS-CoV-2 receptor-binding domain (RBD) antigen formulated with aluminum salt and CpG adjuvants. Hum. Vaccines Immunother..

[33] Jones L.S., Peek L.J., Power J., Markham A., Yazzie B., Middaugh C.R. (2005). Effects of adsorption to aluminum salt adjuvants on the structure and stability of model protein antigens. J. Biol. Chem..

[34] Fox H., Knowlson S., Minor P.D., Macadam A.J. (2017). Genetically Thermo-Stabilised, Immunogenic Poliovirus Empty Capsids; a Strategy for Non-replicating Vaccines. PLoS Pathog..

[35] Cai W., Ping L., Shen W., Liu J., Zhang M., Zhou J., Peng J., Wang M., Zhu Y., Ji G. (2020). Potency of the Sabin inactivated poliovirus vaccine (sIPV) after exposure to freezing temperatures in cold chains. Hum. Vaccines Immunother..

[36] Alt N., Zhang T.Y., Motchnik P., Taticek R., Quarmby V., Schlothauer T., Beck H., Emrich T., Harris R.J. (2016). Determination of critical quality attributes for monoclonal antibodies using quality by design principles. Biologicals.

[37] Campbell J.M., Colombo S., Doyle J.L., Filoti D.I., Hubner G., Magnenat L., Nowinski A.K., Pavon J.A., Singh S.M., Vo L.R. (2024). An Industry Perspective on the use of Forced Degradation Studies to Assess Comparability of Biopharmaceuticals. J. Pharm. Sci..

[38] Basu S., Rustagi R. (2022). Multi-dose vials versus single-dose vials for vaccination: Perspectives from lower-middle income countries. Hum. Vaccines Immunother..

[39] Kraan H., Ten Have R., van der Maas L., Kersten G., Amorij J.P. (2016). Incompatibility of lyophilized inactivated polio vaccine with liquid pentavalent whole-cell-pertussis-containing vaccine. Vaccine.

[40] Sawyer L.A., McInnis J., Patel A., Horne A.D., Albrecht P. (1994). Deleterious effect of thimerosal on the potency of inactivated poliovirus vaccine. Vaccine.

[41] Meyer B.K., Ni A., Hu B., Shi L. (2007). Antimicrobial preservative use in parenteral products: Past and present. J. Pharm. Sci..

[42] Mahmood K., Pelkowski S., Atherly D., Sitrin R.D., Donnelly J.J. (2013). Hexavalent IPV-based combination vaccines for public-sector markets of low-resource countries. Hum. Vaccines Immunother..

[43] Maman K., Zollner Y., Greco D., Duru G., Sendyona S., Remy V. (2015). The value of childhood combination vaccines: From beliefs to evidence. Hum. Vaccines Immunother..

