# Supplementary material for: Three Competitive ELISAs to Quantify the D-Antigen Content of Aluminum-Salt Adjuvanted Recombinant Polio VLPs (Types 1, 2, 3) to Enable Preformulation Characterization Studies
Source: Vaccines (Basel). 2026 May 28;14(6):479. doi: 10.3390/vaccines14060479 (PMC13308473; doi:10.3390/vaccines14060479)
Supplement: Supplementary file 1 [file vaccines-14-00479-s001.zip › vaccines-4277697-supplementary.pdf]

SUPPLEMENTAL INFORMATION

Three Competitive ELISAs to Quantify the D-Antigen Content of Aluminum-Salt Adjuvanted Recombinant Polio VLPs (Types 1, 2, 3) to Enable Preformulation Characterization Studies

Yanli Liu †, John M. Hickey †, Vaskuri G. S. Sainaga Jyothi, Brandy Dotson ‡, Sangeeta B. Joshi and David B. Volkin *

Department of Pharmaceutical Chemistry, Vaccine Analytics and Formulation Center, University of Kansas, 2030 Becker Drive, Lawrence, KS 66047, USA

* Correspondence: volkin@ku.edu; Tel.: +1-785-864-6262; Fax: +1-785-864-5736
† These authors contributed equally to this work.

‡ Current address: Mayo Clinic, 200 First St. SW, Rochester, MN 55905, USA.

Keywords: vaccine; stability; formulation; adjuvant; preservatives; adju-phos; poliovirus; virus-like particles; ELISA; potency; D-antigen

Supplemental Table S1. Linearity and limit of quantification results for monovalent bulk Type 1 PV-VLP. Composition of the monovalent bulk sample was 113-5 DU/mL Type 1 PV-VLP in 10 mM Histidine, 150 mM NaCl, 0.01% PS80 at pH 6.7. Values are presented as mean ± SD (n ≥ 3).

Expected Type 1 DU/mL (% of Nominal)	Monovalent Bulk Type 1 PV-VLP				
	Measured DU/mL (Ave Measured % of Nominal)	SD (DU/mL)	RSD	Accuracy (Measured vs. Expected DU/mL)	n value
113 (125 %)	113 (126 %)	3	2 %	101 %	3
90 (100 %, nominal)	86 (95 %)	5	7 %	95 %	9
68 (75 %)	67 (74 %)	2	3 %	99 %	3
45 (50 %)	42 (47 %)	1	3 %	94 %	3
23 (25 %)	23 (26 %)	3	13 %	104 %	3
18 (20 %)	19 (21 %)	3	12 %	105 %	6
14 (15 %)	15 (17 %)	5	30 %	113 %	6
9 (10 %)	3 (3 %)	5	173 %	30 %	6
5 (5 %)	0 (0 %)	0	-	0 %	3

Supplemental Table S2. Linearity and limit of quantification results for monovalent bulk Type 2 PV-VLP. Composition of the monovalent bulk sample was 20-1 DU/mL Type 2 PV-VLP in 10 mM Histidine, 150 mM NaCl,0.01% PS80 at pH 6.7. Values are presented as mean ± SD (n ≥ 3).

Expected Type 2 DU/mL (% of Nominal)	Monovalent Bulk Type 2 PV-VLP				
	Measured DU/mL (Ave Measured % of Nominal)	SD (DU/mL)	RSD	Accuracy (Measured vs. Expected DU/mL)	n value
20 (125 %)	19 (119 %)	0	3 %	96 %	3
16 (100 %, nominal)	15 (96 %)	1	7 %	97 %	6
12 (75 %)	12 (73 %)	1	7 %	97 %	3
8 (50 %)	7 (45 %)	0	5 %	90 %	3
5 (30%)	4 (24 %)	0	5%	78%	3
4 (25 %)	4 (24 %)	1	15 %	94 %	6
3 (20 %)	3 (20 %)	0	17 %	99 %	6
2 (15 %)	2 (15 %)	0	14 %	102 %	6
2 (10 %)	2 (12 %)	0	12 %	116 %	3
1 (5 %)	2 (10 %)	0	27%	56 %	3

Supplemental Table S3. Linearity and limit of quantification results for monovalent bulk Type 3 PV-VLP. Composition of the monovalent bulk sample was 63-3 DU/mL Type 3 PV-VLP in 10 mM Sodium Phosphate, 150 mM NaCl, 0.01% PS80 at pH 6.8. Values are presented as mean ± SD (n ≥ 3).

Expected Type 3 DU/mL (% of Nominal)	Monovalent Bulk Type 3 PV-VLP				
	Measured DU/mL (Ave Measured % of Nominal)	SD (DU/mL)	RSD	Accuracy (Measured vs. Expected DU/mL)	n value
63 (125 %)	68 (135 %)	2	3 %	108 %	3
50 (100 %, nominal)	55 (109 %)	3	6 %	109 %	3
38 (75 %)	41 (81 %)	2	5 %	108 %	3
25 (50 %)	25 (49 %)	2	6 %	98 %	3
13 (25 %)	12 (23 %)	1	9 %	93 %	6
10 (20 %)	10 (20 %)	1	7 %	98 %	3
8 (15 %)	8 (15 %)	1	7 %	97 %	3
5 (10 %)	5 (9 %)	1	7 %	90 %	3
3 (5 %)	2 (3 %)	2	107 %	65 %	3

Supplemental Table S4. Linearity and limit of quantification results for AP-adjuvanted trivalent PV-VLPs (Types 1, 2, and 3 adsorbed together to AP) using the Type 1 Competitive ELISA. Composition of the formulated trivalent drug product (DP) was 90 DU/mL (Type 1), 16 DU/mL (Type 2), and 50 DU/mL (Type 3) adsorbed together to 0.6 mg/mL AP in 20 mM Histidine, 150 mM NaCl at pH 6.0. AP, aluminum phosphate (Adju-Phos™). Values are presented as mean ± SD (n ≥ 9).

Expected Type 1 DU/mL (% of Nominal)	AP-Adsorbed Trivalent DP (Type 1 PV-VLP)				
	Measured DU/mL (Ave Measured % of Nominal)	SD (DU/mL)	RSD	Accuracy (Measured vs. Expected DU/mL)	n value
113 (125 %)	112 (124 %)	9	8 %	99 %	9
90 (100 %, nominal)	89 (99 %)	6	7 %	99 %	18
68 (75 %)	59 (66 %)	7	12 %	88 %	9
45 (50 %)	41 (45 %)	5	15 %	89 %	9
23 (25 %)	21 (23 %)	4	20 %	91 %	18
18 (20 %)	14 (16 %)	5	41 %	78 %	9
14 (15 %)	10 (11 %)	5	48 %	75 %	9
9 (10 %)	7 (8 %)	5	61 %	80 %	9

Supplemental Table S5. Linearity and limit of quantification results for AP-adjuvanted trivalent PV-VLPs (Types 1, 2, and 3 adsorbed together to AP) using the Type 2 Competitive ELISA. Composition of the formulated trivalent drug product (DP) was 90 DU/mL (Type 1), 16 DU/mL (Type 2), and 50 DU/mL (Type 3) adsorbed together to 0.6 mg/mL AP in 20 mM Histidine, 150 mM NaCl at pH 6.0. AP, aluminum phosphate (Adju-Phos™). Values are presented as mean ± SD (n ≥ 9).

Expected Type 2 DU/mL (% of Nominal)	AP-Adsorbed Trivalent DP (Type 2 PV-VLP)				
	Measured DU/mL (Ave Measured % of Nominal)	SD (DU/mL)	RSD	Accuracy (Measured vs. Expected DU/mL)	n value
20 (125 %)	20 (124 %)	2	8 %	99 %	9
16 (100 %, nominal)	17 (106 %)	2	10 %	106 %	18
12 (75 %)	12 (73 %)	1	8 %	98 %	9
8 (50 %)	8 (50 %)	0	7 %	99 %	9
4 (25 %)	4 (27 %)	0	12 %	108 %	18
3 (20 %)	4 (22 %)	0	11 %	108 %	9
2 (15 %)	3 (16 %)	1	23 %	108 %	9
2 (10 %)	2 (11 %)	0	11 %	110 %	9

Supplemental Table S6. Linearity and limit of quantification results for AP-adjuvanted trivalent PV-VLPs (Types 1, 2, and 3 adsorbed together to AP) using the Type 3 Competitive ELISA. Composition of the formulated trivalent drug product (DP) was 90 DU/mL (Type 1), 16 DU/mL (Type 2), and 50 DU/mL (Type 3) adsorbed together to 0.6 mg/mL AP in 20 mM Histidine, 150 mM NaCl at pH 6.0. AP, aluminum phosphate (Adju-Phos™). Values are presented as mean ± SD (n ≥ 9).

Expected Type 3 DU/mL (% of Nominal)	AP-Adsorbed Trivalent DP (Type 3 PV-VLP)				
	Measured DU/mL (Ave Measured % of Nominal)	SD (DU/mL)	RSD	Accuracy (Measured vs. Expected DU/mL)	n value
63 (125 %)	63 (125 %)	5	7 %	100 %	9
50 (100 %, nominal)	52 (104 %)	6	11 %	104 %	33
38 (75 %)	31 (62 %)	3	8 %	83 %	9
25 (50 %)	22 (43 %)	2	9 %	85 %	9
20 (40 %)	20 (40 %)	3	12 %	99 %	9
18 (35 %)	16 (32 %)	3	18 %	91 %	9
15 (30 %)	13 (26 %)	3	19 %	84 %	9
13 (25 %)	11 (22 %)	3	25 %	89 %	18
10 (20 %)	10 (19 %)	5	48 %	95 %	9
8 (15 %)	6 (12 %)	6	93 %	82 %	9
5 (10 %)	5 (10 %)	6	127 %	95 %	9
